# 

**Published:** 1901-04

**Authors:** 


					﻿SUBSCRIPT low $1 GO^
a ATLANTA PUBLISJidli^ ^ONTHLY.
Burnal-recom
OF MEDICINE. ’I
Successor to Atlanta fledical and Surgical Journal, Established 1855,
and Southern fledical Record, Established 1870.
in.	Atlanta, Ga., April, 1901.	No. 1.

				

